# A reversible lesion of the corpus callosum splenium with adult influenza-associated encephalitis/encephalopathy: a case report

**DOI:** 10.1186/1752-1947-2-220

**Published:** 2008-06-28

**Authors:** En Kimura, Sadahisa Okamoto, Yuji Uchida, Tomoo Hirahara, Tokunori Ikeda, Teruyuki Hirano, Makoto Uchino

**Affiliations:** 1 Department of Neurology, Graduate School of Medical Sciences, Kumamoto University, Honjo, Kumamoto, 860-0811, Japan

## Abstract

**Introduction:**

Influenza virus-associated encephalitis/encephalopathy is a severe childhood illness with a poor prognosis. Adult case reports are rare and, to date, there have been no reports of adults with a mild subcortical encephalopathy with reversible lesions of the corpus callosum splenium.

**Case presentation:**

A previously healthy 35-year-old man presented with acute progressive tetraplegia, transcortical motor aphasia and a mild decrease in his consciousness during his recovery after receiving oseltamivir phosphate treatment, and influenza type A antiviral medication. The initial magnetic resonance imaging study at day 1 showed symmetrical diffuse lesions in the white matter and a lesion on the central portion of the corpus callosum splenium. These findings had resolved on follow-up studies at day 8 and day 146. His neurological deficits mostly recovered within 12 hours following methylprednisolone pulse therapy. The levels of interleukin-6 and interleukin-10 in his blood and cerebrospinal fluid were initially elevated, but rapidly decreased to normal levels by day 8.

**Conclusion:**

It is important for clinicians to recognize that even in adulthood, the subcortical encephalopathy observed during the therapeutic treatment for influenza type A infection can occur in conjunction with a reversible lesion of the corpus callosum, which may recover quickly. In addition, the cytokine storm in the blood system and the corticospinal cavity may play an important role in the etiology of the disease process.

## Introduction

Influenza virus-associated encephalitis/encephalopathy (IAEE) [1-4] is known to have a poor prognosis in childhood, especially in children under the age of 5 years. An acute necrotizing encephalopathy, Reye's syndrome, hemorrhagic shock and encephalopathy are the most feared and often fatal complications in IAEE [5,6]. Although there has been a great improvement in therapeutic approaches, the rates of mortality (31.8%) and disability (27.7%) are still quite high. Recently, the number of patients in Japan with childhood IAEE has increased [3,7], although the number of adult case reports remains small. Pathogenically, IAEE is suggested to be a pro-inflammatory cytokine-related disease [8]. Cytokine levels in serum and cerebrospinal fluid (CSF) are markedly increased in the majority of severe IAEE cases, especially levels of interleukin (IL)-6, IL-10 and soluble tumor necrosis factor receptor 1. Therapeutically, anti-influenza treatments such as a selective neuraminidase inhibitor (oseltamivir phosphate), corticosteroid pulse and hypothermia are quite effective in treating IAEE patients [9]. There are reports demonstrating a variety of magnetic resonance imaging (MRI) findings for IAEE, especially mild cases, such as two children who recovered without any neurological deficit [10]. MRI for these children revealed a lesion in the central portion of the corpus callosum splenium, similar to that of the patient described in this report. In addition, a recent review described such reversible lesions caused by different pathoetiologies [11]. The clinical features of these patients showed relatively mild central nervous system (CNS) manifestations and complete recovery within 1 month.

Here we report the case of an adult patient with mild IAEE, who has recovered without any neurological deficit. The complete follow-up study of MRI and the serum/CSF cytokine assay are presented. MRI revealed a reversible lesion of the central portion of the corpus callosum splenium. Levels of IL-6 and IL-10 in his blood serum and the IL-6 levels in his CSF were initially elevated and later decreased to normal.

## Case presentation

A previously healthy 35-year-old man contracted an influenza type A virus infection. He had a high fever with a mild painful throat, myalgia and arthralgia throughout his whole body. He was diagnosed with an influenza type A infection by a positive result from an influenza antigen detection kit with a sample taken from a throat swab. He started taking oseltamivir phosphate (three 75 mg capsules) within 24 hours of the onset of high fever. The next day, he had an acute progressive tetraplegia and transcortical motor aphasia with mildly altered mental status. He was then transferred to our emergency room for further evaluation.

**Figure 1:**
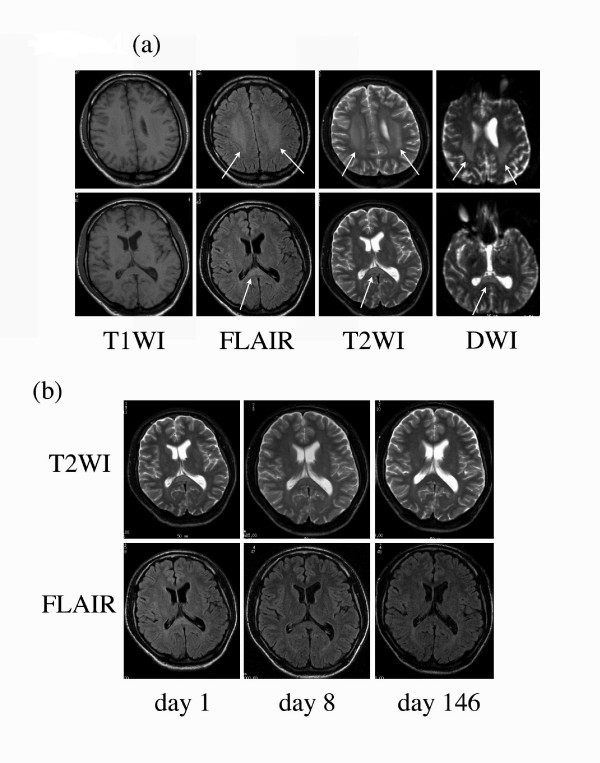
**Magnetic resonance imaging showed a transient signal in the central splenium of the corpus callosum**. (a) Magnetic resonance imaging on day 1: T1-weighted images, fluid-attenuated inversion recovery images, T2-weighted images and diffusion-weighted images. Fluid-attenuated inversion recovery images, T2-weighted images and diffusion-weighted images show lesions in the central splenium of the corpus callosum and symmetric bilateral white matter, but these were not observed in T1-weighted images. (b) The time course of magnetic resonance imaging shows that the lesions in the corpus callosum had resolved, with fluid-attenuated inversion recovery images and T2-weighted images at day 8. Magnetic resonance imaging on day 146 showed that all of these lesions had almost completely disappeared.

Initial MRI at day 1 (Figure 1a) showed lesions diffusely throughout the white matter and especially on the central portion of the corpus callosum splenium, with a slight hyperintensity on T2-weighted fluid-attenuated inversion recovery and markedly high signal intensity on diffusion-weighted images. These findings resolved completely on follow-up study at day 8 and day 146 (Figure 1b).

His cerebrospinal pressure was high (235 mmH_2_O), but cytological and biochemical analyses of CSF were within normal limits: number of cells was 3/3 mm^3 ^(all monocytes), protein level was 30.6 g/dl and the glucose level was 67 mg/dl. The influenza genome was not detected by polymerase chain reaction in CSF samples from day 1 and day 8. Blood count showed a mild thrombocytopenia (12.0 × 10^4 ^cells/ml) and leukopenia (2500 cells/ml). Electroencephalography showed normal basic activity with no paroxysmal discharge. He was treated with methylprednisolone pulse therapy (1000 mg/day) for 3 days; his condition improved quickly following this treatment. After a 2-week rehabilitation, he made a complete recovery and was discharged from the hospital on day 24.

**Figure 2:**
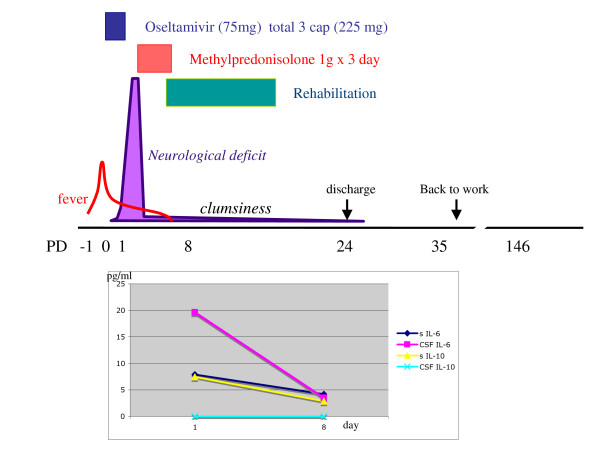
**Clinical course and cytokine levels**. Top, a clinical course of this case is shown. The patient had a high fever with mild painful throat, myalgia and arthralgia. Soon after taking three capsules (225 mg) of oseltamivir phosphate the fever reduced, but was then followed by dullness of consciousness, transcortical motor aphasia and tetraplegia, without sensory disturbance. During corticosteroid pulse therapy all of the neurological deficits disappeared. Bottom, a graph shows the time course of interleukin-6 and interleukin-10 levels in his blood serum and cerebrospinal fluid.

The levels of cytokines in his blood serum and CSF were assayed at pre- and post-treatment with methylprednisolone, as described previously [8]. In the blood serum, IL-6 and IL-10 levels were elevated at day 1 (pre-treatment) and had decreased to normal at day 8 (post-treatment). In the CSF, IL-6 levels were remarkably high (19.6 pg/ml) at day 1 and had decreased to normal by day 8 (Table 1 and Figure 2). The levels of IL-6 and IL-10 in the serum and CSF were correlated with his clinical course.

**Table 1:** Significantly increased interleukin-6 and interleukin-10 levels in the cerebrospinal fluid

	serum		CSF	
day	1	8	1	8
IL-6	**7.9**	**4.2**	**20**	**3.5**
IL-4	3.2	<2.6	<2.6	<2.6
IL-2	<2.6	<2.6	<2.6	<2.6
IFN-γ	<7.1	<7.1	<7.1	<7.1
TNF-α	2.8	<2.8	<2.8	<2.8
IL-10	**7.5**	**2.9**	<2.8	<2.8

				pg/ml

Cytokine assay study of blood serum and cerebrospinal fluid at the time points of pre- and post-treatment with methylprednisolone. In the patient's serum, interleukin-6 and interleukin-10 were elevated at day 1 (pre-treatment) and decreased at day 8 (post-treatment). In his cerebrospinal fluid, interleukin-6 levels were remarkably high (19.6 pg/ml) at day 1 and reduced to normal levels by day 8.

## Discussion

Here, we have presented a case of adult IAEE with transient reversible CNS manifestations and MRI findings revealing a reversible lesion of T2 prolongation and reduced diffusion in the central portion of the corpus callosum splenium. During the initial examination, our first impression of this distinctive MRI finding was an acute disseminated encephalomyelitis (ADEM), which was foremost in our differential diagnosis. Corticosteroid pulse therapy was undertaken and, upon initiation of treatment, the MRI findings rapidly disappeared in conjunction with clinical improvement, although the lesion in the white matter recovered more slowly.

Recently, two cases of children with mild IAEE and with similar MRI findings were reported [10]. These patients developed symptoms soon after the onset of influenza and also had rapid complete recoveries without any permanent functional deficit. The pathogenesis of the reversible lesion in these cases is considered to be due to transient intramyelinic edema. It is postulated that the increased levels of pre-inflammatory cytokines such as IL-6 [3,4,8] might play an important role in the pathogenesis of the lesion. Our cytokine assay, which detected the elevation of the IL-6 and IL-10 levels in our patient's blood serum and CSF, supports this theory. An acute surge in cytokine levels in the blood stream and CSF cavity might trigger vasodilatation following a reversible vasogenic edema of myelin. In addition, similar MRI findings in the central splenium of the corpus callosum have been reported [11] in some cases of infectious encephalitis or encephalopathy other than IAEE, such as rotavirus [12], O-157 *Escherichia coli *[13] and *Salmonella enteritidis *[14]. Despite the different causative agents described in these reports, the clinical manifestations and MRI findings were nearly identical in these cases.

The possibility remains that the oseltamivir phosphate could have aggravated his condition; this selective neuraminidase inhibitor might influence the development of a pathological mechanism that results in vasogenic edema followed by a cytokine storm. It may play a role in aggregating influenza virus particles on the surface of blood cells, endothelial cells or arachnoid cells. These aggregates might then stimulate the release of pro-inflammatory cytokines from these cells. However, we concluded that early treatment with oseltamivir phosphate was still useful in reducing some of his clinical symptoms, including his high fever.

This case was diagnosed as a mild IAEE, and our clinical examinations allowed us to discount other possibly diagnoses, including ADEM and other CNS disorders. The cytokine storm in his blood system and corticospinal cavity played an important role in the pathoetiology of the IAEE.

## Conclusion

We have reported a mild case of IAEE in an adult patient with a transient neurological deficit and interesting reversible lesions in the central splenium of the corpus callosum. It is important for clinicians to recognize that, even in adulthood, the subcortical encephalopathy observed during therapy for influenza type A infection can occur in conjunction with a reversible lesion of the corpus callosum, which may recover quickly. In addition, the cytokine storm in the blood system and the corticospinal cavity may play an important role in the etiology of this disease process.

## List of abbreviations

ADEM: Acute disseminated encephalomyelitis; CNS: Central nervous system; CSF: Cerebrospinal fluid; IAEE: Influenza-associated encephalitis/encephalopathy; IL: Interleukin; MRI: Magnetic resonance imaging.

## Competing interests

The authors declare that they have no competing interests.

## Consent

Written informed consent was obtained from the patient for publication of this case report and any accompanying images. A copy of the written consent is available for review by the Editor-in-Chief of this journal.

## Authors' contributions

EK was the primary physician and neurologist, conceived of the original study, organized and analyzed the data and prepared the draft of the manuscript, SO, YU and ToH were consulting neurologists, evaluated MRI data, assisted with manuscript editing and contributed to the original idea of treating this patient with methylprednisolone, TI performed clinical assessments, TeH was consulted on clinical evaluations and response to therapy, MU organized and analyzed the data, helped to write and edit the case report, and wrote the final draft of the manuscript. All authors read and approved the final manuscript.
